# Genetic links between narcolepsy and ADHD

**DOI:** 10.1038/s41398-021-01420-9

**Published:** 2021-05-28

**Authors:** Drake D. Duane

**Affiliations:** Institute for Developmental Behavioral Neurology/Biological Psychiatry, 8585 East Bell Road, Suite 101, Scottsdale, AZ 85260 USA

**Keywords:** Diagnostic markers, Biomarkers

**Fig. 1:**
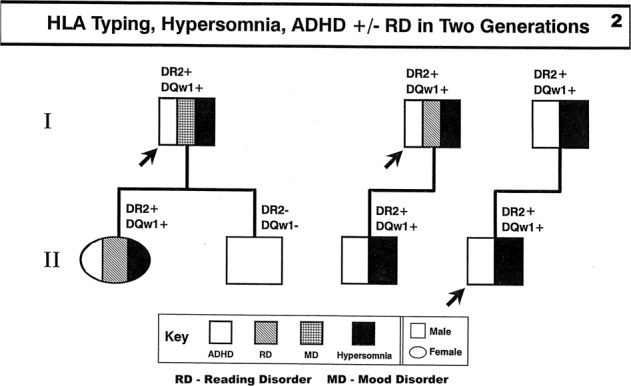
HLA typing using DR-2/DQw-1 markers, hypersomnia, ADHD ± Reading Disorder in two generations.

Dear Editor,

In a recent issue of *Translational Psychiatry*, Takahashi et al.^1^ reported their analysis of shared polygenic risk scores suggest a shared genetic background for both attention deficit hyperactivity disorder (ADHD) and narcolepsy. By another method, we described a similar association between ADHD and narcolepsy. In a paper delivered at the 1995 American Academy of Neurology^2^, we reported three families each in two generations with concurrent ADHD, hypersomnia defined by pupillometry^3^ without sleep log evidence of sleep disorder and both HLA markers DR-2/DQw-1 employed at that time associated with narcolepsy (Fig. 1).

Our subsequent studies have suggested a frequency of HLA markers associated with narcolepsy more than double than anticipated in Caucasians in individuals with behavioral and cognitive evidence of ADHD^4^. Clinically, we have observed late adolescent ADHD patients who will not drive automobiles without their alerting medications for fear of falling asleep. We have additionally observed an increased frequency of these HLA markers as well as hypersomnia in individuals with dyslexia, both with and without ADHD^5^. However, either condition may occur independent of these HLA markers.

The social significance of such an association if valid is that an unknown percentage of ADHD children/adolescents/adults may require alerting medication to become safe adult drivers, as sleepiness increases the risk of motor vehicle accident by a factor of 2.5^6^.
